# Associations of green and blue space exposure in pregnancy with epigenetic gestational age acceleration

**DOI:** 10.1080/15592294.2023.2165321

**Published:** 2023-01-11

**Authors:** Irene Marques, Susana Santos, Giulietta S. Monasso, Serena Fossati, Martine Vrijheid, Mark Nieuwenhuijsen, Vincent W. V. Jaddoe, Janine F. Felix

**Affiliations:** a Generation R Study Group, Erasmus MC, University Medical Center Rotterdam, Rotterdam, the Netherlands; b Department of Pediatrics, Erasmus MC, University Medical Center Rotterdam, Rotterdam, the Netherlands; c EPIUnit - Instituto de Saúde Pública, Universidade do Porto, Porto, Portugal; d Laboratório para a Investigação Integrativa e Translacional em Saúde Populacional (ITR), Universidade do Porto, Porto, Portugal; e ISGlobal, Institute for Global Health, Barcelona, Spain; f Universitat Pompeu Fabra (UPF), Barcelona, Spain; g Spanish Consortium for Research on Epidemiology and Public Health (CIBER), Madrid, Spain

**Keywords:** Green spaces, blue spaces, environmental exposures, DNA methylation, ageing, epigenetic age, gestational age acceleration, pregnancy, cohort study

## Abstract

Early life is seen as a particularly sensitive period for environmental exposures. Natural space exposure during pregnancy has been associated with offspring health. Epigenetic gestational age acceleration, a discrepancy between clinical and DNA methylation-based gestational age, may underlie these associations. In 1359 mother-newborn pairs from the population-based Generation R Study, we examined the associations of natural space exposure, defined as surrounding greenness, distance to major green and blue (water) space, and size of the blue space during pregnancy with offspring epigenetic gestational age acceleration. Natural space exposure was based on participants’ geocoded addresses, and epigenetic gestational age acceleration was calculated from cord blood DNA methylation using Bohlin’s and Knight’s epigenetic clocks. Sensitivity analyses were conducted in a subgroup of newborns with optimal pregnancy dating, based on last menstrual period. Surrounding greenness, measured in normalized difference vegetation index values, was intermediate (median 0.4, IQR 0.2), and 84% and 56% of the participants had a major green or blue space near their home address, respectively. We did not observe associations of natural space availability during pregnancy with offspring epigenetic gestational age acceleration. This could imply that epigenetic gestational age acceleration in cord blood does not underlie the effects of residential natural space availability in pregnancy on offspring health. Future studies could investigate whether residential natural space availability during pregnancy is associated with offspring differential DNA methylation at other CpGs than those included in the epigenetic gestational clocks.

## Introduction

The urban environment has been associated with cardiometabolic health outcomes and mortality in adults, with air pollution being the most frequently studied exposure to date [1,2]. Interest in other measures of the urban environment, such as the availability and proximity of natural spaces, mostly vegetation and water bodies, referred to as green and blue spaces, respectively, has increased in recent years. Green space exposure has been inversely associated with the risk of cardiovascular disease in adults [3,4]. Evidence for associations of blue space with health outcomes is still sparse [5,6]. Early life is a particularly sensitive period for the effects of environmental exposures [7,8], and the exposure to natural space during pregnancy has been associated with beneficial birth outcomes [9–12]. The underlying mechanism for these associations is unclear, but differential DNA methylation might be involved.

DNA methylation has been associated with biological ageing [13,14]. In recent years, multiple epigenetic clocks have been developed, which estimate biological or ‘epigenetic’ age based on DNA methylation levels at a limited number of CpGs [15]. Differences between chronological age and DNA methylation-based age estimates represent epigenetic age acceleration. Positive age acceleration refers to older DNA methylation-based age than chronological age and negative age acceleration refers to younger DNA methylation-based age than chronological age. The first epigenetic clocks were developed for adult age estimation and were based on peripheral blood DNA methylation [13,14]. In adults, positive age acceleration is associated with cardiovascular, cancer, and all-cause mortality [16]. More recently, epigenetic clocks for gestational age at birth have been developed based on cord blood DNA methylation, with those based on the methods by Bohlin and Knight being the most frequently used [17,18]. Several maternal and offspring characteristics have been associated with epigenetic gestational age acceleration, but the directions of effect are inconsistent. For instance, maternal mental diseases and offspring sex have been associated with both positive and negative age acceleration [19–22]; maternal age and maternal BMI where associated with positive gestational age acceleration only in some studies [20–22]; and maternal dietary factors, such as vitamin D3 supplementation or circulating vitamin B12, folate, homocysteine, and fatty acids, show inconsistent associations [23–25]. Thus, a better understanding of how specific exposures are associated with epigenetic gestational age acceleration is needed. In children, a recent study on more than 100 early-life urban environmental exposures did not find associations of green and blue spaces with epigenetic age acceleration at the age of 8 years [26]. However, it is unknown if exposure to natural spaces during pregnancy is associated with epigenetic age acceleration at birth, when there is a shorter period between the exposure and the outcome. We hypothesized that exposure to green or blue space during pregnancy would be associated with epigenetic gestation age acceleration. We did not have a specific hypothesis on the direction of these associations, given the inconsistencies in the previous literature. Therefore, in this study, we aimed to study associations of green and blue space exposure during pregnancy with epigenetic gestational age acceleration based on cord blood DNA methylation.

## Materials and Methods

### Participants

This study was embedded in the Generation R Study, a population-based prospective cohort study from foetal life onwards in Rotterdam, the Netherlands [27]. The Medical Ethical Committee of Erasmus MC, University Medical Center Rotterdam, approved the study (MEC 198.782/2001/31). Pregnant women with an expected delivery date between April 2002 and January 2006 living in Rotterdam were eligible to participate and written informed consent was obtained for all participants. In 1396 of the 9901 live-born newborns participating in the Generation R Study, we measured genome-wide DNA methylation in cord blood. This subgroup was selected from the total study population as a relatively homogeneous, Dutch-ancestry subgroup. Per mother we included only one child, based on completeness of covariates and, if equal, randomly (15 children were excluded based on these criteria). In the current study, we included 1359 mother-child pairs who had information available on cord blood DNA methylation, clinical gestational age at birth, and pregnancy exposure to green and blue spaces.

### Maternal green and blue space exposure during pregnancy

Green and blue space data were generated within the LifeCycle Project framework [28]. A total of eight indicators of natural space were studied in this project. Vegetation index at three distance buffers (100 m, 300 m, and 500 m), distance to major (larger than 5000 m^2^) green space and presence of major green space at less than 300 metres from the home address were used indicators of green space exposure. Distance to major blue space (larger than 5000 m^2^), presence of a major blue space at less than 300 metres from the home address and size of the major blue space were used as indicators of exposure to blue space. Estimated trimester-specific exposures were assigned to each study participant separately for their geocoded addresses through geographic information system platforms.

Normalized Difference Vegetation Index (NDVI) quantifies vegetation by measuring the difference between near-infrared (which vegetation strongly reflects) and red light (which vegetation absorbs). NDVI values range from −1.0 to +1.0. Areas of snow or sand usually show very low NDVI values (for example, 0.1 or less). Sparse vegetation such as grasslands or senescing crops may result in moderate NDVI values (approximately 0.2 to 0.5). High NDVI values (approximately 0.6 to 0.9) correspond to dense vegetation such as that found in temperate and tropical forests or crops at their peak growth stage. Negative values of NDVI (values between −1 and 0) correspond to water and were classified as null. NDVI was derived from the Landsat 4–5 Thematic Mapper (TM), Landsat 7 Enhanced Thematic Mapper Plus (ETM+), and Landsat 8 Operational Land Imager (OLI)/Thermal Infrared Sensor (TIRS). The imagery was selected according to the following criteria: i) cloud cover less than 10%, ii) Standard Terrain Correction (Level 1 T), and iii) greenest period of the year, for best image contrast. In Generation R, NDVI values for pregnancy correspond to Landsat images from 2005, as a reference for the birth years of our population, i.e., 2002–2006, since it was the year with the lowest cloud cover during the recruitment period. Distance, in metres, to the nearest green or blue major space, larger than 5000 m^2^, and size of the respective natural spaces were extracted from the Europe-wide ‘Urban Atlas’ [29].

Pregnancy values were created for all the exposures by calculating an average across the three trimesters. If one trimester value was missing, we used the two known values to calculate the average. If only one trimester value was available, we used that as a proxy of total pregnancy exposure, as the percentage of women moving during pregnancy was relatively low, 7.3%. The variable ‘residential proximity to major green space’ (defined for green spaces in the EU as living within 300 metres of a public open area of more than 5000 m^2^ [30]) was created based on the pregnancy average distance values. Based on previous studies, the same 300 metres cut-off was used to create the variable ‘residential proximity to major blue space’ [26].

### DNA methylation data

DNA samples were extracted from newborn cord blood by the salting-out method. Five hundred nanograms of DNA were bisulphite converted using the EZ-96 DNA Methylation kit (Shallow) (Zymo Research Corporation, Irvine, USA). Samples were processed with the Illumina Infinium HumanMethylation450 BeadChip (Illumina Inc., San Diego, USA). Quality control and normalization were performed using the CPACOR workflow [31]. Probes with a detection *p* ≥ 1E-16 were set to missing. Intensity values were quantile normalized. We removed arrays with technical problems, a call rate ≤95%, or a mismatch between the expected sex of participant and sex determined by chromosome X and Y probe intensities. Probes on the sex chromosomes were removed before the analyses. We used untransformed beta-values as measures of DNA methylation. The final dataset contained information on 458,563 CpGs.

### Epigenetic gestational age

For the primary analyses we used epigenetic gestational age based on Bohlin’s epigenetic clock, calculated with the GAprediction package version 1.16.6 in R. This epigenetic clock predicts epigenetic gestational age based on DNA methylation values of 96 CpGs selected via Lasso regression [17]. In secondary analyses, we used Knight’s epigenetic clock, which estimates epigenetic gestational age based on DNA methylation values of 148 CpGs selected via elastic net regression [18]. The methylclock package 0.5.0 in R was used to calculate raw and residual gestational age acceleration based on Knight’s epigenetic clock. Raw gestational age acceleration (in weeks) is calculated by subtracting clinically estimated gestational age from epigenetic gestational age. Residual gestational age acceleration (in weeks) is calculated as the residuals from the regression of epigenetic gestational age on clinical gestational age. Both raw and residual age accelerations have been previously used in the literature. Raw age acceleration offers a more intuitive representation of the difference between biological and chronological age, whereas residual age acceleration, due to its statistical qualities, corresponds to the component of biological age that is independent of chronological age. Positive gestational age acceleration is defined as older epigenetic gestational age than clinical gestational age, and negative gestational age acceleration is defined as younger epigenetic gestational age than clinical gestational age.

### Clinical gestational age

Pregnant women were seen for foetal ultrasound at a dedicated research centre at the first study visit. During this visit, we established a clinical gestational age. If mothers had a known and reliable first day of the last menstrual period, and a regular menstrual cycle of 28 ± 4 days, this estimate was based on their last menstrual period, what we consider optimal pregnancy dating. If mothers did not know the exact date of their last menstrual period, or had an irregular menstrual cycle, we established the gestational age by ultrasound [32]. Clinical gestational age at birth was retrieved from midwife or obstetric records.

### Covariates

Potential covariates were selected based on previous literature. Maternal covariates included age at intake, education level, categorized into low and medium education versus higher education, parity, as nulliparous versus multiparous, smoking during pregnancy, divided into no smoking and quitting when pregnancy was known versus sustained smoking, and neighbourhood deprivation index, based on the Dutch deprivation index and categorized in tertiles [33]. This index is calculated based on residents’ characteristics, such as education, income, and job market position. Child sex was also included as a covariate. Maternal information was obtained via questionnaires sent out in each pregnancy trimester. Information on child sex and birth weight was obtained from midwife and hospital records. Cord blood cell-type proportions were obtained from the ‘Salas’ reference panel for the estimation of cell-type proportion in the ‘FlowSorted.CordBlood.Combined.450 K’ Bioconductor package [34]. This reference set includes the following cell types: CD8+ T cells, CD4+ T cells, natural killer cells, B cells, monocytes, granulocytes, nucleated red blood cells. Covariate missing values (up to a maximum of 8% for maternal smoking) were imputed using the Markov chain Monte Carlo method, and pooled analysis was conducted from five imputed datasets [35].

### Statistical analysis

We determined correlations between clinical and epigenetic gestational age based on the Bohlin and Knight methods using Spearman’s correlation coefficients. Correlation between exposures was tested with pairwise Spearman correlation tests. A non-response analysis compared the newborns included in the analyses to those who participated in Generation R but who did not have DNA methylation measured through chi-square tests, Student’s t-tests, and Mann–Whitney tests. Outcome distributions were inspected using histograms (**Supplemental Figure 1**). Non-linear associations of natural spaces with age acceleration were ruled out by visual inspection of scatterplots and, when in doubt, with generalized additive models. We used linear regression models to examine associations of green and blue space availability during pregnancy with raw and residual gestational age acceleration. Standardized NDVI was assessed per IQR change, distance to major green and blue space in 1-kilometre increments, and blue space size in 1 square kilometre increments. Gestational age acceleration calculated based on Bohlin’s epigenetic clock was used in primary analysis due to its higher correlation with clinical gestational age. Knight’s clock was used in secondary analyses. The crude model was adjusted for child sex and batch effects, by including plate number. The main model was additionally adjusted for maternal age, education, parity and smoking, neighbourhood deprivation index, and estimated cell type proportions. To examine the impact of variation in cell-type proportions, the main model was also analysed without cell-type adjustment (reduced main model) [22]. We also planned models with additional adjustment for birth weight and air pollution, based on atmospheric particulate matter of less than 2.5 micrometres, to explore the roles of these factors in any significant associations from the primary models. In an additional analysis, we excluded preterm births (clinical gestational age <37 weeks) and repeated the primary and secondary analyses in the main group. Sensitivity analyses were performed in the subset of mothers with optimal pregnancy dating based on last menstrual period (total N = 376). We accounted for multiple testing by dividing the nominal *p* values by two, to consider the two categories of exposure being tested, i.e., green and blue spaces, as the specific exposures within those categories are correlated. Therefore, *p* values ≤0.025 were considered significant. All analyses were performed in Statistical Package for the Social Sciences version 25.0 (SPSS IBM, Chicago, Illinois, United States).

## Results

### Participant characteristics

**Figure 1.**
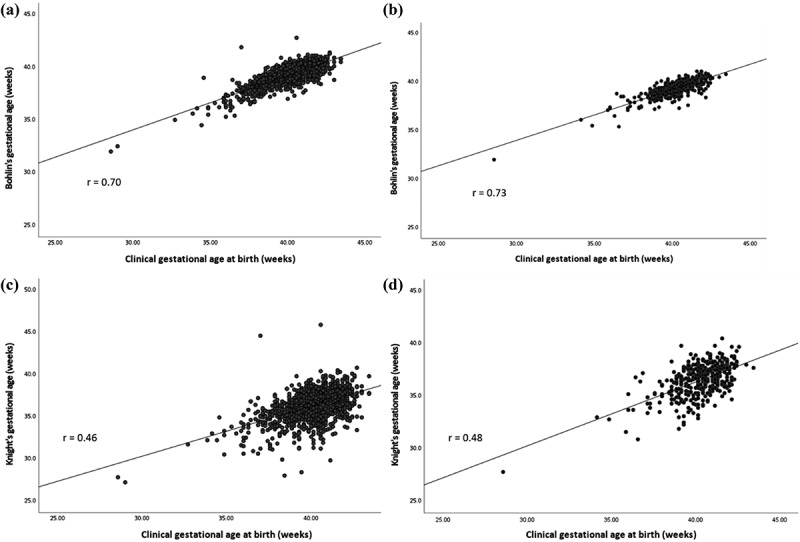
Spearman pairwise correlation between clinical and epigenetic gestational age. a) and b) estimated by Bohlin’s epigenetic clock in full group newborns (N = 1359) and in subgroup newborns with optimal pregnancy dating (N = 376), respectively. c) and d) estimated by Knight’s epigenetic clock in full group newborns and in subgroup newborns with optimal pregnancy dating, respectively.

We included 1359 mother-newborn pairs from the Generation R Study with information on natural space exposure during pregnancy and cord blood DNA methylation. Table 1 shows the participant characteristics before imputation of covariates (Supplemental Table 1 shows participant characteristics after imputation). NDVI values were intermediate (median 0.4, IQR 0.2) across all the buffer distances, which corresponds to sparse vegetation or grass landscapes. Supplemental Table 2 shows correlation matrix between exposures. Overall, NDVI showed a weak positive correlation with distance to blue space and weak inverse correlations with having a blue space at 300 metres from the address and with the size of the blue space. Clinical gestational age (mean 40.2 weeks, SD 1.5) was on average older than epigenetic gestational age based on Bohlin’s epigenetic clock (39.3 weeks, SD 1.0). This was reflected in both raw and residual gestational age acceleration, which had negative mean values. In the full study group, Spearman’s correlation between clinical and epigenetic gestational age was r = 0.70, very similar to the correlation in the subgroup of mothers with optimal clinical pregnancy dating (r = 0.73) (Figure 1). Epigenetic gestational age based on Knight’s epigenetic clock was younger (36.3, SD 1.7) and Spearman’s correlation with clinical gestational age was lower than that obtained for Bohlin’s method, r = 0.46 in the full study group and r = 0.48 in the optimal clinical pregnancy dating subgroup (Figure 1).

**Table 1.** Maternal and newborn characteristics based on non-imputed data (n = 1359).

**Maternal Characteristics**	
Age at intake (years)	31.7 (4.2)
Pre-pregnancy body mass index (kg/m^2^)	24.2 (4.0)
Education	
No or primary	25 (1.8%)
Secondary	442 (32.5%)
Higher	872 (64.2%)
Parity	
Nulliparous	824 (60.6%)
Multiparous	533 (39.2%)
Smoking	
Non-smoker or smoked until pregnancy was known	1069 (78.7%)
Smoked throughout pregnancy	179 (13.2%)
Neighbourhood deprivation index in tertiles	
[1] low deprived	351 (25.8%)
[2] medium deprived	371 (27.3%)
[3] high deprived	633 (46.6%)
NDVI 100 m buffer	0.4 (0.3–0.5)
NDVI 300 m buffer	0.4 (0.3–0.5)
NDVI 500 m buffer	0.4 (0.4–0.5)
Major green space at 300 m (yes)	1137 (83.7%)
Distance to major green space (m)	146.5 (72.3, 248.5)
Major blue space at 300 m (yes)	762 (56.1%)
Distance to major blue space (m)	268.6 (127.1, 429.6)
Size of major blue space with (m^2^)	23,918.9 (13,403.0, 64,974.4)
PM_2.5_	20.4 (18.1, 22.6)
**Newborn Characteristics**	
Sex (girl)	668 (49.2%)
Birth weight (grams)	3548 (511)
Gestational age at birth (weeks)	40.2 (1.5)
Epigenetic gestational age (Bohlin, weeks)	39.3 (1.0)
Raw gestational age acceleration (Bohlin, weeks)	−0.9 (0.9)
Epigenetic gestational age (Knight, weeks)	36.3 (1.7)
Raw gestational age acceleration (Knight, weeks)	−3.8 (1.6)

Values are mean (SD) or median (1^st^ quartile, 3^rd^ quartile) for continuous variables and counts (%) for categorical variables. NDVI, normalized difference vegetation index. Missing values: maternal smoking, 111; maternal education, 20; maternal BMI, 8; neighbourhood deprivation index, 4; parity,2

Non-response analysis showed that included participants had older mothers, who had lower body mass indexes and were more highly educated. Participants lived in less deprived neighbourhoods and were closer to both green and blue spaces than non-participants (Supplemental Table 3).

### Associations of green and blue spaces with epigenetic gestational age acceleration

We did not observe associations of any of the eight indicators of green and blue space availability during pregnancy with offspring epigenetic gestational age acceleration based on either Bohlin’s or Knight’s epigenetic clocks. Sensitivity analyses conducted on newborns with optimal pregnancy dating followed the same patterns as the main analyses (Table 2). Further models planned with additional adjustment for birth weight and air pollution were not conducted, due to lack of associations in the main model. Exclusion of preterm births (gestational age <37 weeks) did not materially change the results (data not shown).

**Table 2.** Associations of residential green and blue space in pregnancy and epigenetic gestational age acceleration based on Bohlin and Knight’s epigenetic clock in the full population (N = 1359) and sensitivity group (N = 376).

	Full population (N = 1359)				Subgroup with optimal pregnancy dating (N = 376)			
	Raw Age Acceleration ^a^		Residual Age Acceleration ^b^		Raw Age Acceleration ^a^		Residual Age Acceleration ^b^	
**Bohlin’s Epigenetic Clock**	Difference (95% CI)	*p*	Difference (95% CI)	*p*	Difference (95% CI)	*p*	Difference (95% CI)	*p*
NDVI 100 m	0.04 (−0.04, 0.11)	0.31	−0.02 (−0.06, 0.03)	0.42	−0.11 (−0.24, 0.03)	0.13	−0.03 (−0.12, 0.05)	0.42
NDVI 300 m	0.03 (−0.17, 0.05)	0.45	−0.03 (−0.08, 0.02)	0.24	−0.09 (−0.24, 0.06)	0.24	−0.04 (−0.13, 0.05)	0.42
NDVI 500 m	0.05 (−0.03, 0.13)	0.25	−0.03 (−0.08, 0.02)	0.31	−0.06 (−0.22, 0.09)	0.43	−0.04 (−0.13, 0.05)	0.37
Green space at 300 m	−0.11 (−0.03, 0.24)	0.11	0.05 (−0.03, 0.13)	0.25	0.13 (−0.12, 0.38)	0.31	0.04 (−0.11, 0.19)	0.61
Distance to green space	−0.17 (−0.54, 0.21)	0.38	−0.05 (−0. 27, 0.18)	0.68	−0.09 (−0.76, 0.57)	0.78	−0.13 (−0.53, 0.26)	0.51
Blue space at 300 m	−0.01 (−0.11, 0.09)	0.89	0.02 (−0.04, 0.08)	0.49	0.18 (−0.02, 0.37)	0.08	0.05 (−0.07, 0.17)	0.40
Distance to blue space	0.12 (−0.01, 0.34)	0.20	0.01 (−0.11, 0.14)	0.86	−0.19 (−0.60, 0.23)	0.37	−0.09 (−0.34, 0.16)	0.58
Blue space size	< −0.01 (< −0.01, <0.01)	0.86	< −0.01 (< −0.01, <0.01)	0.13	< −0.01 (−0.10, <0.01)	0.80	< 0.01 (−0.10, <0.01)	0.90
**Knight’s Epigenetic Clock**								
NDVI 100 m	0.02 (−0.09, 0.13)	0.67	−0.02 (−0.12, 0.08)	0.70	−0.12 (−0.31, 0.06)	0.20	−0.07 (−0.23, 0.10)	0.41
NDVI 300 m	0.04 (−0.08, 0.16)	0.54	−0.01 (−0.22, 0.51)	0.90	−0.02 (−0.23, 0.19)	0.85	0.02 (−0.16, 0.20)	0.79
NDVI 500 m	0.04 (−0.09, 0.17)	0.56	−0.02 (−0.13, 0.09)	0.73	0.01 (−0.21, 0.22)	0.96	0.03 (−0.16, 0.21)	0.79
Green space at 300 m	0.04 (−0.16, 0.25)	0.69	−0.01 (−0.19, 0.17)	0.95	−0.04 (−0.38, 0.30)	0.83	−0.12 (−0.42, 0.18)	0.43
Distance to green space	−0.06 (−0.63, 0.51)	0.84	−0.02 (−0.48, 0.52)	0.93	0.21 (−0.70, 1.12)	0.64	0.12 (−0.60, 0.99)	0.63
Blue space at 300 m	−0.01 (−0.16, 0.14)	0.91	0.01 (−0.12, 0.15)	0.83	0.21 (−0.05, 0.48)	0.12	0.12 (−0.12, 0.35)	0.34
Distance to blue space	0.09 (−0.25, 0.42)	0.61	−0.01 (−0.31, 0.28)	0.94	−0.22 (−0.79, 0.35)	0.44	−0.16 (−0.65, 0.34)	0.54
Blue space size	< −0.01 (< −0.01, <0.01)	0.43	< −0.01 (< −0.01, <0.01)	0.81	< −0.01 (−0.10, <0.01)	0.55	< −0.01 (−0.10, <0.01)	0.37

Values represent regression coefficients (95% confidence interval) and reflect the difference in raw and residual gestational age acceleration at birth per increase of 1 interquartile range for NDVI, 1 kilometre for green and blue space distances, and 1 square kilometre for blue space size. Results are based on the main models, which were adjusted for maternal age, education, parity and smoking, child sex, batch effects (by including plate number), and estimated cell proportions.

NDVI, normalized difference vegetation index; CI, confidence interval

^a^Raw gestational age acceleration (in weeks) was obtained by subtracting the clinical estimate of gestational age from DNA methylation gestational age

^b^Residual gestational age acceleration (in weeks) was calculated from the residuals from a regression model of DNA methylation gestational age on clinical gestational age

## Discussion

In this study, we examined the associations between residential green and blue space exposure during pregnancy and epigenetic gestational age acceleration at birth. We did not find evidence of associations between the indicators of natural space availability during pregnancy with epigenetic gestational age acceleration at birth measured in cord blood in 1359 participants in the Generation R. This was the case for both clocks used to estimate epigenetic gestational age acceleration, as well as when restricting the sample to offspring of women who had optimal pregnancy dating based on a regular and known date of last menstrual period.

Previous studies showed associations between natural space exposure during pregnancy and birth outcomes [9–12], but the underlying mechanism is still not known. DNA methylation was found to be associated with residential greenness in adults [36,37]. Epigenetic gestational age acceleration may underlie the associations for birth outcomes in children, and we hypothesized that residential exposure to natural space during pregnancy would be associated with cord blood epigenetic gestational age acceleration at birth. We did not find evidence of associations between green and blue space availability during pregnancy with epigenetic gestational age acceleration based on cord blood DNA methylation.

Studies assessing associations between natural space exposure in early life and markers of biological ageing are scarce. A previous study that looked into several urban exposures and epigenetic age acceleration in childhood also did not find any associations with either green or blue space exposure [26]. Although we hypothesized that a shorter period between the exposure and the assessment of age acceleration could reveal an association, our findings are in line with this previous study. A study in Iranian children, where average NDVI values were much lower than in our population, identified positive associations of green space exposure in preschool children and telomere length, which is used as an ageing marker [38]. Jointly, these studies suggest that natural space exposure in early life might be associated with biological ageing, but they do not provide evidence to support that this is reflected in epigenetic (gestational) age acceleration.

The epigenetic clocks used in this analysis may capture aspects of biological ageing that do not reflect *in-utero* adaptation to environmental exposures, but this does not exclude an association of residential natural space with differential DNA methylation at other CpGs or in other tissues. Bohlin’s epigenetic clock performed better in our population than Knight’s clock, based on the correlation between estimated and clinical gestational ages (Spearman correlations in the full group were 0.70 and 0.46, respectively). This was expected due to the similarities in terms of European ancestry and clinical gestational age ranges between our cohort and that used by Bohlin in the epigenetic clock development. Still, the consistent findings obtained using different epigenetic clock methodologies suggest that the null findings are not dependent on the selected methodology. We found similar effect sizes and directions of effect using two different epigenetic gestational age calculation methods. The majority of exposures show consistent direction of effects between the clocks, especially in the full population. This indicates that both clocks may be capturing similar biological ageing processes. Similar results in the full study group and subgroup of mothers with pregnancy dating based on a regular menstrual cycle indicate that the null findings are likely not explained by inaccurate pregnancy dating in the full group. However, the increased precision of pregnancy dating in this group may have been outweighed by the fact that the sample size was much smaller, thus decreasing power. We expected relatively small effect sizes for natural space exposure. Maternal smoking is one of the strongest exposures in relation to differential DNA methylation at birth [39]. In the current literature, only one study found an association of maternal smoking with residual epigenetic age acceleration, with an effect size of 0.09 weeks for smoking versus non-smoking [20]. We expected the effect sizes in this study to be smaller than that. Prado et al. [26] found a non-significant association of NDVI at 100 m with child epigenetic age of −0.02 weeks (CI −4.87,5.3), in line with our findings. Miri et al. [38] examined the associations of natural space exposure with telomere length and showed effect sizes ranging from −21.8% to 8.3% for distance to major green space and home address NDVI at 300 m, respectively. As the telomere length is a related, but different outcome, a direct comparison of effect sizes with the latter study is not possible. However, both shorter distances to major green spaces and higher NDVI at 300 m were associated with greater telomere length (i.e., younger biological age). In contrast, in our analyses of residual age acceleration estimated by Bohlin’s clock, a shorter distance to major green space was associated with older biological age. However, the results for NDVI at 300 m being associated with younger biological age were in line with the paper by Miri et al. However, our results were non-significant and should be carefully interpreted.

Strengths of the present study include data collected from a large and well-established prospective birth cohort, detailed and precise information on residential green and blue space exposure and DNA methylation, and the possibility to conduct sensitivity analyses in a subgroup with optimal pregnancy dating. NDVI is the most common method to access surrounding greenness and this allows direct comparison to other relevant studies. The inclusion of additional indicators of natural spaces, such as distance and accessibility to major green and blue space are also strengths of this study.

However, our study also has limitations. First, NDVI does not reflect all the relevant aspects and types of green areas, for example, if it is an attractive and accessible area [40]. Second, in the urban setting of the Generation R Study, we might have limited variation in the exposure to green and blue spaces, which is reflected in the intermediate NDVI values at different distances and also in the proximity to major green and blue spaces characterizing our population. Despite the relatively large sample size, this might limit the detection of associations with small effect sizes. Third, the exposure assessment is limited to the geocoded residential address and may not represent true exposure or time spent in or near the natural space area. However, a recent study compared different methodologies of urban exposure assessment and concluded that methods based only on residential address obtained very similar results to those accounting for time spent outside of residential location [41]. Fourth, environmental exposures might have trimester-specific effects. However, the method used in these analyses calculates one value per address per year. Therefore, we could in theory only examine trimester-specific results in women who changed address during pregnancy, which was 7.3% of our population, leaving a sample size too low for meaningful analyses. Fifth, this study was conducted in a European ancestry and highly educated subgroup of the Generation R Study, which may limit the generalizability of the findings to other ethnicities and socioeconomic groups. Fifth, we adjusted our association models for several measured confounders. As in any observational study, residual confounding may play a role. However, as our findings were null, we do not consider it likely that this would have had a major impact on our study. Future studies are needed to confirm our findings and to look at the relation between green and blue space exposure in early life, epigenetic ageing, and child health outcomes.

## Conclusion

This study found no evidence to support associations of residential green and blue space availability during pregnancy with epigenetic gestational age acceleration at birth. This might imply that epigenetic gestational age acceleration in cord blood estimated by Bohlin or Knight’s epigenetic clocks does not underlie effects of residential natural space in pregnancy on offspring health, but our findings need further confirmation. Future studies could focus on larger populations with optimal pregnancy dating or investigate if residential natural spaces during pregnancy are associated with differential DNA methylation at other CpGs.

## Supplementary Material

Supplemental Material

## Data Availability

Data described in the manuscript are available from the corresponding author on reasonable request, subject to the Generation R Study data access procedures.

## Abbreviations:

CpGs cytosine-guanine dinucleotide sites

IQR interquartile range

NDVI normalized difference vegetation index

SD standard deviation
