# Supplementary material for: Associations of green and blue space exposure in pregnancy with epigenetic gestational age acceleration
Source: Epigenetics. 2023 Jan 11;18(1):2165321. doi: 10.1080/15592294.2023.2165321 (PMC9980449; doi:10.1080/15592294.2023.2165321)
Supplement: Supplemental Material [file KEPI_A_2165321_SM8974.docx]

## Suplementary Tables

**Supplemental table 1** - Maternal and child characteristics based on non-imputed and imputed data (N=1359).

| **Maternal Characteristics** | Non-imputed data | Imputed data |
| --- | --- | --- |
| Age at intake (years) | 31.7 (4.2) | 31.7 (4.2) |
| Pre-pregnancy body mass index (kg/m^2^) | 24.2 (4.0) | 24.2 (4.0) |
| Education |  |  |
| No or primary and secondary | 467 (34.3%) | 475 (35.0%) |
| Higher | 872 (64.2%) | 884 (65.0%) |
| Parity |  |  |
| Nulliparous | 824 (60.6%) | 825 (60.7%) |
| Multiparous | 533 (39.2%) | 534 (39.3%) |
| Smoking |  |  |
| Non-smoker or smoked until pregnancy was known | 1069 (78.7%) | 1162 (85.5%) |
| Smoked throughout pregnancy | 179 (13.2%) | 197 (14.5%) |
| Neighborhood deprivation index in tertiles |  |  |
| (1) low deprived | 351 (25.8%) | 352 (25.9%) |
| (2) medium deprived | 371 (27.3%) | 373 (27.4%) |
| (3) high deprived | 633 (46.6%) | 634 (46.7%) |
| **Newborn Characteristics** |  |  |
| Sex (girl) | 668 (49.2%) | 668 (49.2%) |
| Birth weight (grams) | 3548 (511) | 3548 (511) |
| Gestational age at birth (weeks) | 40.2 (1.5) | 40.2 (1.5) |

Values are mean (SD) or median (1^st^ quartile, 3^rd^ quartile) for continuous variables and counts (%) for categorical variables. NDVI, normalized difference vegetation index.

**Supplemental table 2** - Correlation matrix of exposure variables

|  | Green Space | | | | | Blue Space | | |
| --- | --- | --- | --- | --- | --- | --- | --- | --- |
| Green Space | NDVI 100 | NDVI 300 | NDVI 500 | Distance | at 300m | Distance | at 300m | Size (m2) |
| NDVI 100 | 1,00 | 0.81 | 0.71 | -0.36 | 0.21 | 0.19 | -0.14 | -0.05 |
| NDVI 300 |  | 1,00 | 0.94 | -0.33 | 0.28 | 0.10 | -0.07 | -0.04 |
| NDVI 500 |  |  | 1,00 | -0.26 | 0.25 | 0.06 | -0.05 | -0.02 |
| Distance |  |  |  | 1,00 | -0.78 | -0.75 | 0.07 | 0.09 |
| at 300m |  |  |  |  | 1,00 | 0.05 | -0.03 | -0.06 |
| Blue Space |  |  |  |  |  |  |  |  |
| Distance |  |  |  |  |  | 1,00 | -0.86 | 0.04 |
| at 300m |  |  |  |  |  |  | 1,00 | -0.03 |
| Size (m2) |  |  |  |  |  |  |  | 1,00 |

Values correspond to Spearman correlation and are based on pairwise comparison.

**Supplemental table 3** - Non-response analysis

|  | Analysis group | Non-response group |  |
| --- | --- | --- | --- |
| **Maternal Characteristics** | (N=1359) | (N=7290) | *p* value |
| Age at intake (years) | 31.7 (4.2) | 29.5 (5.5) | < 0.01 |
| Pre-pregnancy body mass index (kg/m^2^) | 24.2 (4.0) | 25.0 (4.7) | < 0.01 |
| Education |  |  | < 0.01 |
| No or primary | 25 (1.8%) | 844 (11.6%) |  |
| Secondary | 442 (32.5%) | 3114 (42.7%) |  |
| Higher | 872 (64.2%) | 2262 (31.0%) |  |
| Parity |  |  | < 0.01 |
| Nulliparous | 824 (60.6%) | 3847 (52.8%) |  |
| Multiparous | 533 (39.2%) | 3109 (42.6%) |  |
| Smoking |  |  | < 0.01 |
| Non-smoker or smoked until pregnancy was known | 1069 (78.7%) | 4904 (67.3%) |  |
| Smoked throughout pregnancy | 179 (13.2%) | 1172 (16.1%) |  |
| Neighborhood deprivation index in tertiles |  |  | < 0.01 |
| (1) low deprived | 351 (25.8%) | 804 (11.0%) |  |
| (2) medium deprived | 371 (27.3%) | 1342 (18.4%) |  |
| (3) high deprived | 633 (46.6%) | 5124 (70.3%) |  |
| NDVI 100m buffer | 0.4 (0.3 - 0.5) | 0.4 (0.3, 0.4) | < 0.01 |
| NDVI 300m buffer | 0.4 (0.3 - 0.5) | 0.4 (0.3, 0.5) | < 0.01 |
| NDVI 500m buffer | 0.4 (0.4 - 0.5) | 0.4 (0.3, 0.5) | < 0.01 |
| Major green space at 300m (yes) | 1137 (83.7%) | 6829 (79.0%) | < 0.01 |
| Distance to major green space (m) | 146.5 (72.3, 248.5) | 290.3 (93.6, 279.9) | < 0.01 |
| Major blue space at 300m (yes) | 762 (56.1%) | 4530 (52.4%) | < 0.01 |
| Distance to major blue space (m) | 268.6 (127.1, 429.6) | 290.3 (144.2, 489.3) | < 0.01 |
| Size of major blue space (m^2^) | 23918.9 (13403.0, 64974.4) | 30362.5 (16600.3, 62666.9) | < 0.01 |
| PM_2.5_ | 20.4 (18.1, 22.6) | 19.6 (17.9, 21,6) | < 0.01 |
| **Newborn Characteristics** |  |  |  |
| Sex (girl) | 668 (49.2%) | 3615 (49.6%) | 0.77 |
| Birth weight (grams) | 3548 (511) | 3379.4 (565.1) | 0.02 |
| Gestational age at birth (weeks) | 40.2 (1.5) | 40.0 (39.0, 40.9) | <0.01 |

Values are mean (SD) or median (1^st^ quartile, 3^rd^ quartile) for continuous variables and counts (%) for categorical variables. NDVI, normalized difference vegetation index.

The analysis compared included participants (N=1359) to those with exposure information who participated in Generation R at birth but where not included in the analyses (N=7290) because DNA methylation was not measured (N= 8107) or their sibling was included (N=15). Twins (N=232) and siblings (N=570) were excluded from non-response population.

Differences between groups were calculated by independent sample t-tests (normally distributed continuous variables), Mann Whitney test (non-parametric continuous variables) and chi-square testes (categorical variables).

## Suplementary Figures


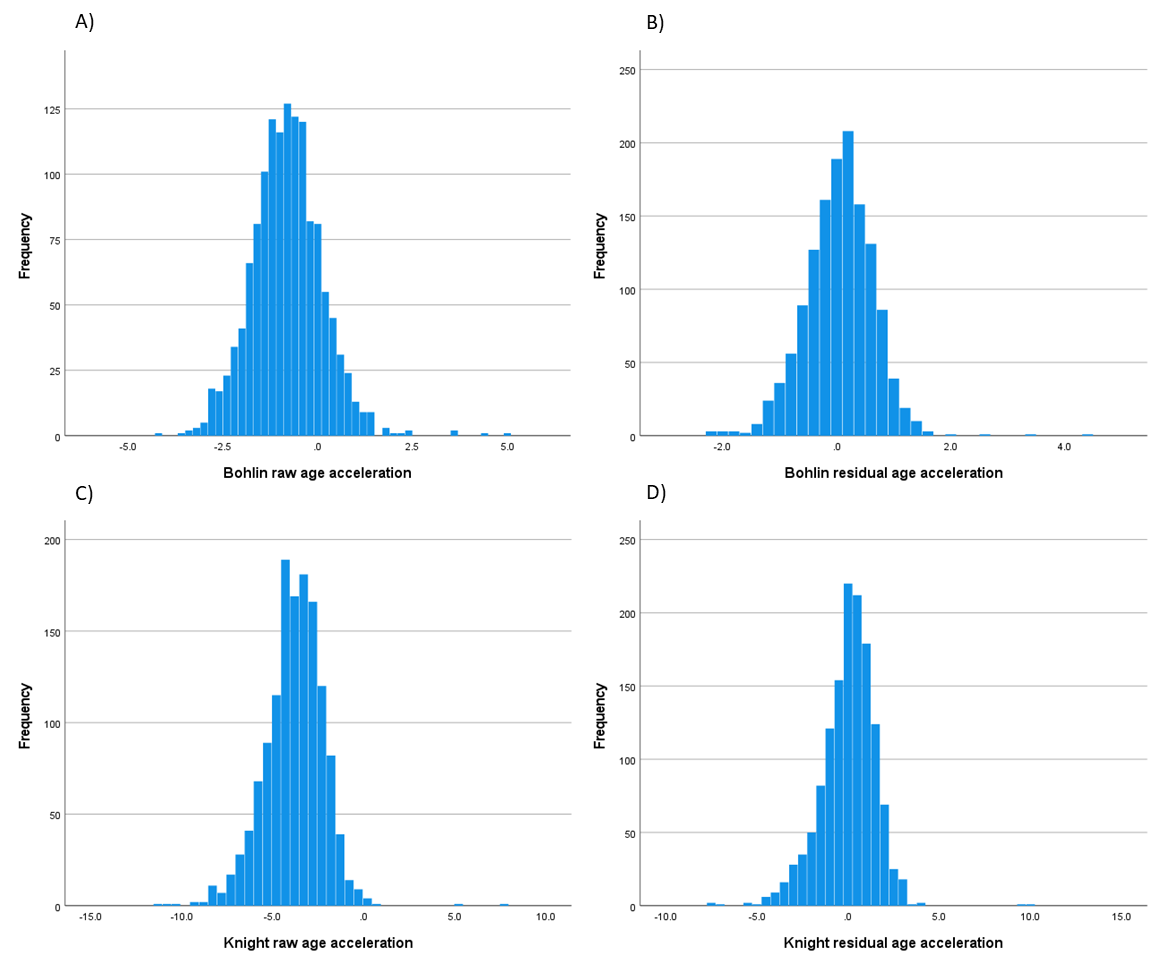


**Supplemental Figure 1** – Histograms of the outcomes: A) Bohlin raw age acceleration; B) Bohlin residual age acceleration; C) Knight raw age acceleration; D) Knight residual age acceleration.
